# Inhalable Vaccines: Can They Help Control Pandemics?

**DOI:** 10.3390/vaccines10081309

**Published:** 2022-08-13

**Authors:** Vivek P. Chavda, Lalitkumar K. Vora, Vasso Apostolopoulos

**Affiliations:** 1 Department of Pharmaceutics and Pharmaceutical Technology, L M College of Pharmacy, Ahmedabad 380009, Gujarat, India; 2 School of Pharmacy, Queen’s University Belfast, 97 Lisburn Road, Belfast BT9 7BL, UK; 3 Immunology and Translational Research Group, Institute for Health and Sport, Victoria University, Melbourne, VIC 3030, Australia; 4 Immunology Program, Australian Institute for Musculoskeletal Science (AIMSS), Melbourne, VIC 3021, Australia

**Keywords:** pulmonary vaccine, intranasal vaccine, inhalable vaccine, SARS-CoV-2, COVID-19, vaccine, IgA, mucosal immunity

## Abstract

The emergence of a new coronavirus presents a huge risk to public health worldwide and has spread widely amongst the human population. Since its emergence, the severe acute respiratory syndrome-coronavirus-2 (SARS-CoV-2) is frequently evolving by mutation and genetic recombination to give rise to new viral variants. These emerging variants pose a challenge to existing COVID-19 management strategies and vaccine efficacy. Interruption of viral spread is required as the merging variants pose higher transmissibility than the previous ones. To achieve this, local protection of the respiratory tract with immunity is essential. Here, we advocate the use of pulmonary/inhalable vaccines to achieve this goal.

Since the emergence of “severe acute respiratory syndrome-coronavirus-2 (SARS-CoV-2)” in December 2019, immunization continues to be the foundation of healthcare today [1,2]. Though anti-SARS-CoV-2 vaccines are being produced at a record rate, all approved or licensed vaccines are parenteral products requiring strictly controlled frozen systems. The stringent storage conditions and requirements of trained healthcare personnel impede vaccine accessibility and delivery to lower-income countries [3,4]. The SARS-CoV-2 virus enters the human body by airborne droplets and direct contact. The virus travels to the rear of the nasal passageways, where it attaches to and enters host cells via the heterodimeric, angiotensin-converting enzyme-2 (ACE-2) on the membrane of bronchial epithelial cells [5] even though, more recently, it has been reported that SARS-CoV-2 has other entry points into host cells [6,7,8,9]. From there, the virus travels through the mucosa of the pharynx and respiratory passages before entering the lungs and infecting pneumocytes of type 2 pulmonary epithelia [10]. Severe infection can lead to “acute respiratory distress syndrome (ARDS)”, where there is a rise in pro-inflammatory cytokines and subsequent widespread lung inflammation [11,12]. In regards to SARS-CoV-2 vaccinations, antibodies are generated, which are safeguarded against COVID-19 [13]. The intramuscular injection of the marketed vaccines induces robust serum IgG levels to safeguard the lower respiratory system but does not provoke epithelial IgA immune responses (in respiratory fluids and serum) that can defend the upper respiratory tract. IgA may access the upper respiratory tract via the mucociliary pathway, but only when circulating IgG levels are elevated [14,15,16]. Epithelial cells function as sensors that identify any introduction of infectious material through pattern-recognition receptors and convey signals to primary mucosal cells to activate non-specific, innate defenses and increase adaptive immunity [17]. “Pathogen-associated molecular patterns (PAMPs, e.g., through toll-like receptors) or damage-associated molecular patterns (DAMPs, e.g., from infected cells) stimulate antigen uptake by antigen-presenting cells (APCs).” Multiple effectors are involved in mucosal immune responses, including secretory IgA antibodies, mucosal cytotoxic T cells and mucosal IgG that is generated locally or acquired from the serum. Secretory IgA serves as the mucosa’s initial line of defense by blocking infection entrance. Outer host defenses that impede microbe entrance at mucosal surfaces also hinder the effective uptake of mucosally administered vaccinations [18]. Therefore, it is essential to determine which mucosally administered antigen formulations may elicit robust and long-lasting B and T cell responses (Figure 1) [19]. Multiple cases, such as intranasal inactivated or attenuated influenza virus vaccines, the oral polio vaccine, and oral attenuated Salmonella Typhi and Vibrio cholera vaccines, among many others against numerous enteric and respiratory infections, confirm the efficacy of this approach [20]. Upon infection through the nasal mucosa, stimulation of IgA levels via the mucosal immune system results [21,22]. Cytokines such as IL-12 and granulocyte/macrophage colony-stimulating factor (GM-CSF), or a cocktail of cytokines, may boost mucosal immune responses upon vaccination [17]. In addition, mucosal dendritic cells are able to migrate and transport antigen to systemic inductive sites such as the lymph nodes and spleen. Activated CD8^+^ T cells in response to mucosal antigens may initially exhibit a rather unconstrained migratory pattern, but with time, memory CD8^+^ T cells demonstrate a preference for the tissue in which antigen was first met. Similarly, IgG is essential for protective immunity in the lower respiratory tract, but IgA is more critical in the nasal region [14,23]. Vaccination at mucosal locations minimizes the likelihood of “antibody-dependent disease enhancement (ADE)” by preventing viral entry at the mucosal site. It has often been demonstrated that injection of mucosal vaccination induces robust systemic humoral immunity, eliminating any virus particle that evades the main immune response at the mucosal site [24,25]. In addition to IgA, a small proportion of generated antibodies include mainly secretory IgM, which is likewise reliant on the pIgR and IgD antibodies.

Therefore, there seems to be an unrelenting need to create inhalation-based immunization strategies that follow the similar process of COVID-19; start of infection (viral entry), advancement (viral shedding), and aggravation. Intranasal vaccines are known to prevent airborne diseases by providing multifront immunization effectively.

While COVID-19 has encouraged the development of innovative vaccination formulations, a major fraction of the proteinaceous compositions presented practical difficulties with respect to temperature control. Investigation of inhaled vaccine administration should be carried out on the creation of stable dry vaccine dosage forms, which have the benefit of improved stability and may thus be kept for longer durations without decomposition at less stringent temperatures. Aerosolized vaccinations should be carefully explored as a means of combating highly contagious viral infections such as COVID-19 [26]. The Ad5-nCoV based dry inhalation vaccine is also licensed in a phase 1/2 clinical trial with 840 participants (NCT04840992). A worldwide phase 3 clinical study is ongoing to determine the effectiveness, immunogenicity and safety of Cansino and Beijing Institute of Biotechnology-manufactured Ad5-nCoV in healthy human volunteers (aged 18 and older) (NCT04526990). The phase 1/2 clinical investigation of COVID-19 vaccine candidate MRT5500 is also in progress with randomized, double-blind, placebo-controlled methods to rule out the safety, reactogenicity (tolerance), and immunogenicity (immune response). It is anticipated that a total of 415 healthy participants aged 18 or older will be recruited for the study among 13 experimental locations [27]. A recent study noted that a single inhalation dose of ChAd-SARS-CoV-2-S elicited substantial levels of antibodies in pre-clinical mouse studies. This might enhance T cell and mucosal IgA defenses, hence limiting SARS-CoV-2 entry and multiplication in the nasal airway and alveolar area [28]. The steerable adenovirus type 5 vaccination against SARS-CoV-2 elicited a robust response in terms of the production of mucosal IgA, CD4^+^ CD8^+^ T cells and complementing serum neutralizing antibodies [29]. A phase III multicenter clinical study in randomized, double-blind, parallel-controlled mode, with approximately 13,000 participants aged 18 and older who have previously received one intramuscular injection of Ad5-nCoV will be conducted. Volunteers must have had an injectable Ad5-nCoV vaccination >56 days previous to participation. All participants will receive one dose of the experimental vaccination or a placebo by nebulized inhalation. The ratio of individuals in the placebo group to the vaccine group is 1:1, and the experimental vaccine’s effectiveness and safety will be monitored for 52 weeks following inoculation (NCT05124561).

We believe that the spectrum of vaccination development should be expanded to include aerosolized dry powder compositions due to the many benefits of dry vaccines (particularly inhalable vaccinations, which may provide extra protection by stimulating IgA-mediated mucosal immune responses). Its dryness may significantly improve the stability and longevity of vaccines. Nano/microparticulate forms of vaccines may be breathed using portable inhalers or nasal spray, eliminating the requirement for skilled medical staff and simplifying mass immunization programs [26]. Research on comparable coronaviruses shows that mucosal vaccination may induce durable systemic and mucosal immunity to protect against COVID-19. In fact, the creation of ‘universal’ mucosal vaccines containing homologous antigens on influenza and coronaviruses may be a realistic strategy to preventing future pandemics (notwithstanding its difficulty) [23].

## Figures and Tables

**Figure 1:**
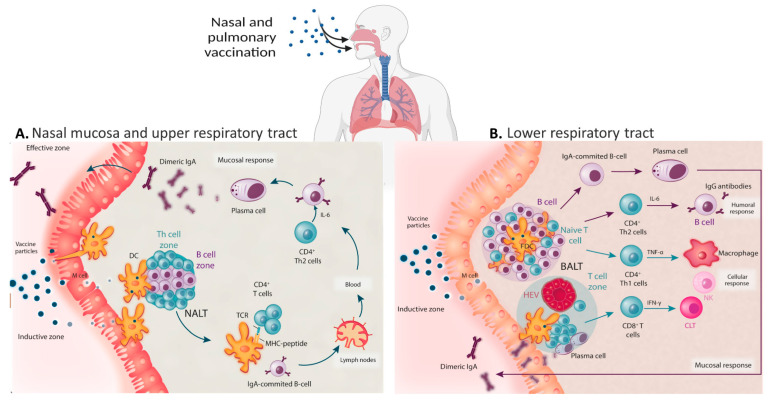
Mechanism of action and series of events following pulmonary vaccination; (**A**) Protective immune responses in the nasopharynx-associated lymphoid tissue (NALT), with the pathogen-mediated reaction stemming mainly from secretory IgA antibodies produced by mucosal epithelial cells. (**B**) Humoral immune response in the lower respiratory tract with bronchus-associated lymphoid tissue (BALT) stimulates humoral and mucosal/local immune responses. Abbreviations: DC, dendritic cell; NK, natural killer; TCR, T cell receptor; CTL, cytotoxic T lymphocyte. Some of the objects created using biorender.com.

## Data Availability

Not applicable.
